# Histomorphometric Evaluation of Non-Thermal Plasma-Treated Xenogenic Bone Graft for Enhanced Bone Regeneration in a Rabbit Calvarial Defect Model

**DOI:** 10.3390/jfb17060280

**Published:** 2026-06-05

**Authors:** Hyunsuk Choi, Yong-Suk Moon, Hyung-Gyun Kim, Dong-Seok Sohn

**Affiliations:** 1 Department of Dentistry and Prosthodontics, Daegu Catholic University School of Medicine, Daegu 42472, Republic of Korea; hschoi@cu.ac.kr; 2 Department of Anatomy, Daegu Catholic University School of Medicine, Daegu 42472, Republic of Korea; ysmoon@cu.ac.kr; 3 Department of Dentistry and Advanced General Dentistry, Daegu Catholic University School of Medicine, Daegu 42472, Republic of Korea; hgkim25@cu.ac.kr; 4 Department of Dentistry and Oral and Maxillofacial Surgery, Daegu Catholic University School of Medicine, Daegu 42472, Republic of Korea

**Keywords:** non-thermal plasma treatment, xenograft, bone regeneration, histomorphometric analysis, rabbit calvarial defect

## Abstract

When placing dental implants, xenografts are most commonly used clinically to compensate for the insufficient bone volume of patients. However, xenografts have limitations including low osteoinductive capacity and prolonged healing time. This study aimed to determine whether non-thermal plasma treatment could enhance the regenerative performance of bovine cancellous bone graft (SANTA-OSS^®^) in a rabbit calvarial defect model. Twenty-four adult male New Zealand white rabbits received bilateral 8 mm critical-size calvarial defects. One defect was filled with untreated SANTA-OSS (control) and the contralateral defect with plasma-treated SANTA-OSS using the ACTILINK™ Reborn device. Animals were sacrificed at 2, 4, and 8 weeks (*n* = 8 per group) for histomorphometric analysis. The plasma-treated group showed significantly higher new bone area (14.12 ± 0.69%, 18.93 ± 0.68%, and 32.72 ± 0.61% at 2, 4, and 8 weeks) than the control at all time points (*p* < 0.05). In addition, the experimental group exhibited accelerated graft resorption, larger bone marrow area, greater blood vessel area, and more TRAP-positive osteoclasts compared with the control (*p* < 0.05). Within the limitations of this study, non-thermal plasma treatment significantly enhanced new bone formation and promoted favorable graft remodeling, while also accelerating graft resorption, increasing bone marrow area, and improving vascularization. These findings suggest that simple chairside plasma activation can improve the regenerative performance of xenografts.

## 1. Introduction

Dental implants have been a widely used treatment for lost teeth [1]. However, in cases of alveolar bone defects caused by various factors such as periodontal disease, inflammation, or alveolar bone atrophy, the width or height of the bone is often insufficient for implant placement [2]. In such cases, alveolar bone grafting is frequently used in dental clinical practice, either simultaneously with or prior to implant placement [3]. In addition, bone augmentation using graft materials is required not only for dental implant placement but also for the reconstruction of large cystic or tumor defects, for pre-prosthetic or orthodontic site development, and for promoting bone healing in mandibular fractures involving the ramus, body, or symphysis regions [4].

The purpose of alveolar bone graft materials is to induce and promote the regenerative capacity of alveolar bone. Bone regeneration is broadly categorized into osteogenesis, osteoinduction, and osteoconduction [5]. Osteogenesis is the process of new bone formation and development, occurring when the cells within the grafted bone material themselves proliferate bone. Osteoinduction is a method that induces bone regeneration by utilizing bone-forming proteins or other growth factors that trigger osteoinduction stimulation. Osteoconduction is a method in which the bone graft material fills the bone defect, acting as a connecting structure for bone formation [6,7].

Autologous bone graft materials possess all three of the aforementioned mechanisms and have long been regarded as the gold standard for dental bone graft treatment due to their advantages of excellent biocompatibility and freedom from immune reactions. However, autologous bone has drawbacks, such as the need for additional donor sites, risks of trauma or infection during harvesting, limited harvesting volume, and potential pain at the harvesting site and a prolonged healing period [8].

Allogeneic and xenogeneic bone grafts have also been widely used as alternatives to autologous bone grafts. These materials have the advantages of being readily available in large quantities, eliminating the need for additional surgery at the donor site, and maintaining structural stability as a scaffold to fill bone defects. Allografts are generally known to contain higher levels of intrinsic growth factors and bone morphogenetic proteins (BMPs) than xenografts, resulting in superior osteoinductive properties, faster bone remodeling rates, and greater advantages in maintaining long-term bone volume. However, in the oral environment, saliva and the oral microbiome can negatively affect the integration of the graft, and allografts may be more vulnerable to bacterial contamination and immune responses than xenografts. Among them, bovine-derived cancellous bone xenografts, which possess a structure similar to human bone, are the most frequently used in clinical practice. However, these xenografts have limitations, including low osteoinductive capacity, a risk of immune reactions or infection, and the need for a long healing period before they are completely replaced by new bone formation [9,10].

To overcome the limitations of existing xenograft materials, various surface modification technologies have been studied to enhance the biological performance of the grafts [11]. Recently, in the field of dental clinical practice, the method of treating implant surfaces or alveolar bone graft materials with non-thermal atmospheric pressure plasma has been attracting attention [12]. Plasma, the fourth state of matter, is created by applying high voltage to gases such as O_2_, Ar, and N_2_ to generate reactive oxygen and nitrogen species. When this plasma is applied to the surface of bone graft materials or titanium implants, it can effectively remove hydrocarbon contaminants accumulated during the biological aging process [12]. Furthermore, as the contact angle approaches 0°, superhydrophilicity is restored, facilitating the adsorption of proteins such as fibronectin and albumin, and significantly enhancing cell adhesion, proliferation, and differentiation [11,12]. These modifications promote the initial bone formation process, increase alkaline phosphatase (ALP) activity, and stimulate angiogenesis and new bone formation without altering the original surface morphology of the graft material [12,13].

Previous in vitro and in vivo studies have shown that treating the surface of implant fixtures with plasma improves blood compatibility and reduces oxidative stress, thereby accelerating osseointegration [14]. Furthermore, results indicate that plasma treatment prevents the stability dip phenomenon—where implant stability decreases after placement—allowing for a rapid recovery of stability [15].

While there has been some research on plasma surface treatment for implants as described above [14,15], its application to xenografts remains limited. Studies on plasma-treated bone grafts are scarce, and comprehensive histomorphological evaluations in standardized defect models are still lacking [16]. Since bone graft materials are continuously exposed to saliva and oral microorganisms in the oral cavity, non-thermal plasma treatment is expected to reduce bacterial adhesion and make the graft less susceptible to the adverse effects of the oral environment.

Therefore, the aim of this study was to evaluate the effects of non-thermal plasma-treated bovine cancellous bone graft on new bone formation, graft resorption, bone marrow formation, and vascularization in a rabbit calvarial defect model. This study represents Part I of a companion series, in which histomorphometric analysis was performed. The immunohistochemical analysis of osteogenic and angiogenic markers will be reported separately in Part II.

## 2. Materials and Methods

### 2.1. Experimental Materials

In this study, bovine-derived cancellous bone graft (SANTA-OSS^®^, BIOTEM Co., Ltd., Hanam-si, Gyeonggi-do, Republic of Korea) and the ACTILINK™ Reborn non-thermal atmospheric pressure plasma device (Plasmapp Co., Ltd., Daejeon, Republic of Korea) were used. Plasma activation of the SANTA-OSS surface was performed immediately before implantation using the ACTILINK™ Reborn device. Each graft underwent two consecutive 1 min treatment cycles according to the manufacturer’s protocol [17]. This chairside plasma treatment effectively removes surface hydrocarbon contaminants and restores superhydrophilicity without altering the original topography of the graft particles.

### 2.2. Surgical Procedures

**Figure 1:**
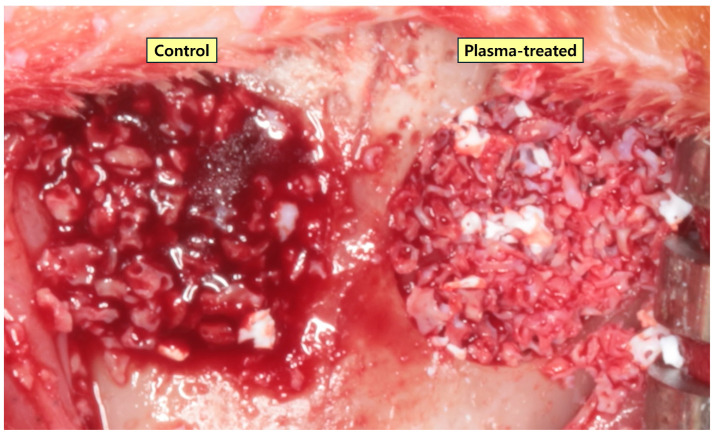
Application of bone graft materials in the rabbit calvarial defects. Left: untreated SANTA-OSS (Control group); Right: non-thermal plasma-treated SANTA-OSS (Experimental group).

Twenty-four adult male New Zealand white rabbits weighing 2.8–3.2 kg (average 3.0 kg) were used. All experimental procedures were approved by the Institutional Animal Care and Use Committee of Daegu Catholic University Medical Center (approval number: DCIAFCR-240620-12-Y). The rabbits were randomly assigned to three healing-period groups (2, 4, and 8 weeks; *n* = 8 per group). General anesthesia was induced with an intramuscular injection of ketamine (30 mg/kg, Ketalar; Yuhan Co., Seoul, Republic of Korea) and xylazine (10 mg/kg, Rompun; Bayer Korea, Seoul, Republic of Korea). Additionally, 0.5 mL of lidocaine with 1:100,000 epinephrine was injected subcutaneously along the midline of the calvaria. After skin and periosteal incision along the sagittal midline, the surgical site was irrigated with sterile saline. No additional antibacterial solution was used on the calvaria. Two circular critical-size full-thickness defects (8 mm in diameter, penetrating the entire thickness of the calvaria until the dura mater was exposed) were created in the frontal bone using an 8 mm trephine burr. The resected bone disks were carefully removed without damaging the underlying dura. The side (left or right) receiving the plasma-treated SANTA-OSS was randomly assigned using a computer-generated randomization table prior to surgery to minimize any side-related bias. In each animal, one defect was filled with 0.5 cc of untreated SANTA-OSS (control group), and the contralateral defect was filled with 0.5 cc of plasma-treated SANTA-OSS (experimental group) (Figure 1). The critical-size defects were filled exclusively with the SANTA-OSS. No additional blood soaking, blood-clot technique, platelet-rich fibrin (PRF), or platelet-rich plasma (PRP) was used. All animals were housed in standardized laboratory conditions with the same commercial diet, controlled temperature (22 ± 2 °C), humidity (50 ± 10%), and a 12 h light/dark cycle throughout the experimental period. The periosteum and skin were sutured with 4-0 nylon (Blue nylon, Ailee Co., Busan, Republic of Korea). All animals received intramuscular gentamicin (20 mg/kg, Donghwa Co., Seoul, Republic of Korea) for 3 days postoperatively. The rabbits were sacrificed at 2 weeks (*n* = 8), 4 weeks (*n* = 8), or 8 weeks (*n* = 8) after surgery.

### 2.3. Tissue Preparation

At the designated time points (2, 4, and 8 weeks), the experimental animals were sacrificed under general anesthesia. They were euthanized by an intravenous overdose of pentobarbital sodium while under general anesthesia, in accordance with institutional animal care guidelines and the humane euthanasia recommendations of the American Veterinary Medical Association (AVMA). The calvarial specimens were harvested using a microsaw. Harvested specimens were immediately fixed in 10% neutral buffered formalin for 24 h at room temperature, washed with 0.1 M phosphate-buffered solution, and decalcified in 10% formic acid for 10 days. After decalcification, the specimens were embedded in paraffin (Paraplast^®^; Sigma-Aldrich Co., Oxford, MS, USA) and serially sectioned at 5 μm thickness through the center of each defect. The sections were stained with hematoxylin-eosin (H&E) and Masson’s trichrome (MT) for histological evaluation of new bone formation and soft tissue changes.

### 2.4. Histomorphometric Measurements

Ten randomly selected fields from each specimen were photographed at ×20 and ×200 magnification using an Axiophot photomicroscope (Carl Zeiss; Jena, Germany) equipped with an AxioCam MRc5 camera (Carl Zeiss; Jena, Germany). Histomorphometric measurements were performed using AxioVision SE64 software (Carl Zeiss, Germany). The following parameters were quantified as percentages of the total augmented area:

- Newly formed bone area;
- Remaining graft material area;
- Soft tissue area;
- Bone marrow area;
- Blood vessel area.

The total augmented area included newly formed bone, residual graft particles, fibrous/soft tissue, bone marrow, and vascular structures within the original 8 mm defect boundaries. All histomorphological measurements were performed by a single experienced examiner who was unaware of the group assignment (control group vs. experimental group) and healing period to minimize observer bias.

### 2.5. Tartrate-Resistant Acid Phosphatase (TRAP) Staining Procedure

Tartrate-resistant acid phosphatase (TRAP) activity was detected using an acid phosphatase kit (Sigma-Aldrich, St. Louis, MO, USA) according to the manufacturer’s instructions. Paraffin-embedded sections were cleared, dehydrated, and incubated for 1 h at 37 °C in the dark with a mixture containing naphthol AS-BI phosphate (25 mg), fast garnet GBC salt (15 mg), and 27 mmol tartaric acid in 0.1 mol acetate buffer (pH 5.2). The sections were counterstained with acid hematoxylin and mounted. Slides were examined under an Axiophot photomicroscope with AxioVision SE64 software. For each slide, twenty fields were randomly selected, and the number of TRAP-positive multinucleated osteoclasts (dark brown) was counted in a standardized 1 mm^2^ area of the augmented region. The counting of TRAP-positive multinucleated osteoclasts was performed by the same blinded examiner as described in Section 2.4.

### 2.6. Statistical Analysis

The sample size was determined using G*Power software (version 3.1). Based on previous studies on rabbit calvarial defects, the effect size of 1.5 was assumed for the percentage of newly formed bone area, with α = 0.05 and power (1 − β) = 0.80. As a result, a minimum of 6 experimental animals were required per group; therefore, statistical power was secured in this study by using 8 animals per healing period, taking into account the dropout rate.

Data are expressed as mean ± standard error. Statistical analyses were performed using SPSS software (version 25.0, IBM Corp., Chicago, IL, USA). Differences between the control and experimental groups at each time point, as well as differences among healing periods within each group, were evaluated by one-way analysis of variance (ANOVA) followed by Tukey’s post hoc test. A *p*-value < 0.05 was considered statistically significant.

## 3. Results

### 3.1. Histological Analysis

SANTA-OSS particles were lightly stained and clearly distinguished from the surrounding tissues in both hematoxylin-eosin (H&E) and Masson’s trichrome (MT) stains. Lamellar host bone stained red, whereas woven or newly formed bone stained blue in the MT stain. No bacteria or signs of inflammation were observed in any histological section either the control or experimental group under light microscopy. In all specimens, the center of the defect remained level without depression, and the SANTA-OSS particles maintained the space-maintaining property.

**Figure 2:**
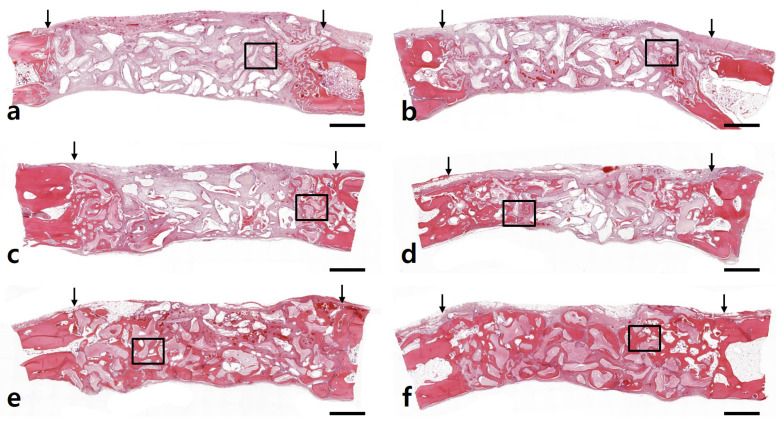
Low-magnification images (×20) of the rabbit calvaria after surgery at 2 weeks (**a**), 4 weeks (**c**), and 8 weeks (**e**) in the control group, and at 2 weeks (**b**), 4 weeks (**d**), and 8 weeks (**f**) in the experimental group. The arrows indicate the margins of the defect. The boxed areas are presented at higher magnification in Figure 3. Hematoxylin-eosin stain (Scale bar: 1000 μm).

**Figure 3:**
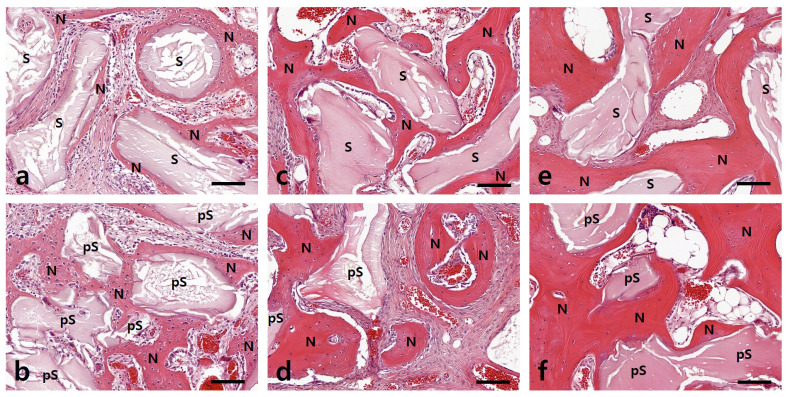
Higher-magnification images (×200) showing the new bone formation after surgery at 2 weeks (**a**), 4 weeks (**c**), and 8 weeks (**e**) in the control group, and at 2 weeks (**b**), 4 weeks (**d**), and 8 weeks (**f**) in the experimental group. N, newly formed bone; S, SANTA-OSS particles; pS, plasma-treated SANTA-OSS particles. Hematoxylin-eosin stain (Scale bar: 100 μm).

**Figure 4:**
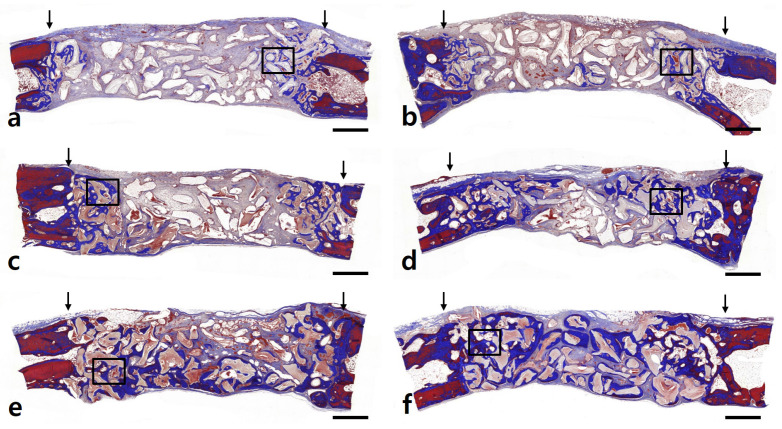
Low-magnification images (×20) of the rabbit calvaria after surgery at 2 weeks (**a**), 4 weeks (**c**), and 8 weeks (**e**) in the control group, and at 2 weeks (**b**), 4 weeks (**d**), and 8 weeks (**f**) in the experimental group. The arrows indicate the margins of the defect. The boxed areas are presented at higher magnification in Figure 5. Masson’s trichrome stain (Scale bar: 1000 μm).

**Figure 5:**
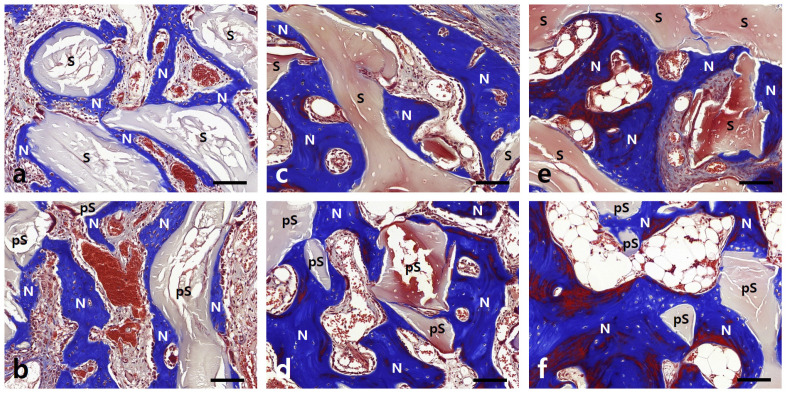
Higher-magnification images (×200) showing the new bone formation after surgery at 2 weeks (**a**), 4 weeks (**c**), and 8 weeks (**e**) in the control group, and at 2 weeks (**b**), 4 weeks (**d**), and 8 weeks (**f**) in the experimental group. N, newly formed bone; S, SANTA-OSS particles; pS, plasma-treated SANTA-OSS particles. Masson’s trichrome stain (Scale bar: 100 μm).

At 2 weeks, new bone formation was limited to the defect margins in both groups, although the amount of newly formed bone differed markedly between groups. By 4 weeks, the thickness and density of newly formed bone had increased compared with the 2-week specimens. At 8 weeks, abundant new bone was observed along the defect margins and around the graft particles in both groups (Figure 2, Figure 3, Figure 4 and Figure 5).

In the control group, newly formed bone at 2 weeks was confined to the defect margins and graft surfaces, with osteoblasts lining the newly formed bone (Figure 2a, Figure 3a, Figure 4a and Figure 5a). At 4 weeks, bone thickness and density had increased, yet bone formation remained predominantly at the margins, while graft particle density decreased (Figure 2c, Figure 3c, Figure 4c and Figure 5c). At 8 weeks, new bone on graft surfaces had further increased, but only a small amount reached the defect center; bone marrow spaces began to appear between new bone and residual graft particles (Figure 2e, Figure 3e, Figure 4e, and Figure 5e).

In the experimental group (plasma-treated SANTA-OSS), new bone formation at 2 weeks was already observed extending toward the defect center. Newly formed bone covered the surfaces of plasma-treated graft particles and extended into the intervening connective tissue, with numerous osteoblasts present (Figure 2b, Figure 3b, Figure 4b, and Figure 5b). At 4 weeks, bone thickness and density were markedly greater than at 2 weeks, with some new bone reaching the defect center; notably, numerous large and small blood vessels were evident within the connective tissue (Figure 2d, Figure 3d, Figure 4d, and Figure 5d). At 8 weeks, new bone thickness and density were substantially higher than at 4 weeks. Graft particle size and density had decreased further, and prominent bone marrow spaces containing adipose tissue were observed between the newly formed bone trabeculae (Figure 2f, Figure 3f, Figure 4f, and Figure 5f).

### 3.2. Histomorphometric Analysis

**Figure 6:**
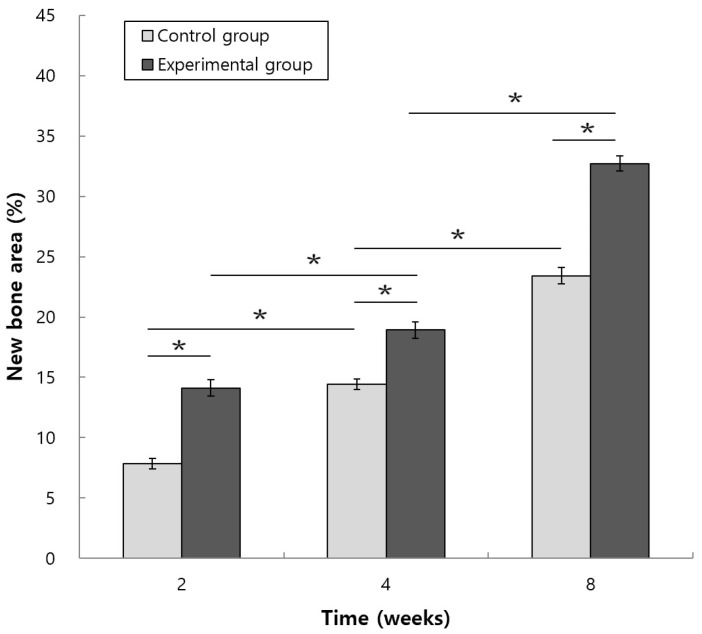
Histomorphometric measurement of the area of newly formed bone to the area of the total augmented area at 2, 4, and 8 weeks in the control group and experimental group. (* *p* < 0.05).

The percentage of newly formed bone area relative to the total augmented area in the control group was 7.85 ± 0.46%, 14.44 ± 0.44%, and 23.41 ± 0.68% at 2, 4, and 8 weeks, respectively. In the experimental group, the corresponding values were 14.12 ± 0.69%, 18.93 ± 0.68%, and 32.72 ± 0.61%. One-way ANOVA with Tukey’s post hoc test revealed that new bone area at 8 weeks was significantly greater than at 2 and 4 weeks in both groups (*p* < 0.05). Furthermore, the percentage of newly formed bone was significantly higher in the experimental group than in the control group at all time points (Figure 6).

**Figure 7:**
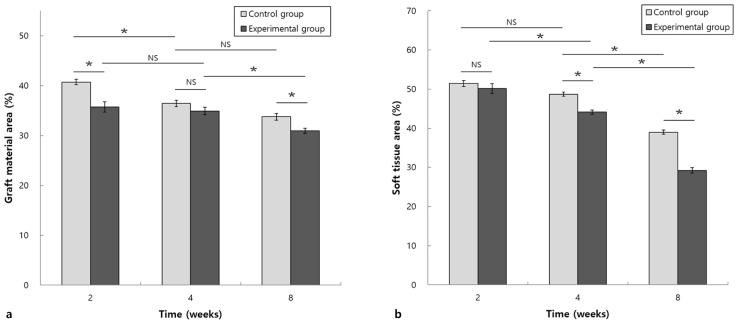
Histomorphometric measurement of the area of (**a**) remaining graft material and (**b**) soft tissue to the total augmented area at 2, 4, and 8 weeks in the control and experimental group. (* *p* < 0.05; NS, not significant).

The percentage of remaining graft material area in the control group was 40.72 ± 0.53%, 36.42 ± 0.64%, and 33.77 ± 0.67% at 2, 4, and 8 weeks, respectively. In the experimental group, the values were 35.71 ± 1.04%, 34.90 ± 0.75%, and 30.93 ± 0.53%. Graft material area at 8 weeks was significantly lower than at 2 weeks in both groups (*p* < 0.05). The remaining graft area was significantly smaller in the experimental group at 2 and 8 weeks compared with the control group (Figure 7a).

The percentage of soft tissue area in the control group was 51.43 ± 0.76%, 48.70 ± 0.55%, and 39.00 ± 0.55% at 2, 4, and 8 weeks, respectively. In the experimental group, the values were 50.17 ± 1.21%, 44.15 ± 0.53%, and 29.23 ± 0.71%. Soft tissue area at 8 weeks was significantly lower than at 2 and 4 weeks in both groups (*p* < 0.05). The soft tissue area was significantly smaller in the experimental group at 4 and 8 weeks compared with the control group (Figure 7b).

**Figure 8:**
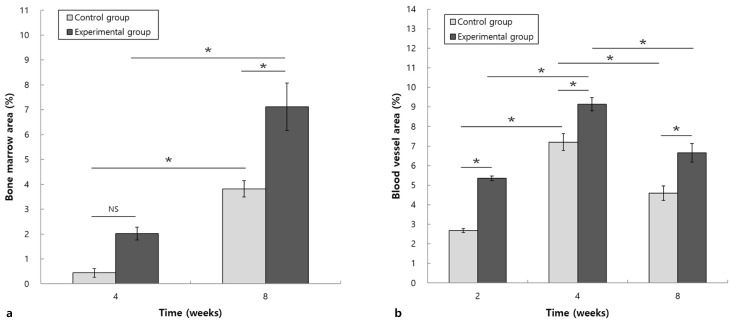
Histomorphometric measurement of the area of (**a**) bone marrow and (**b**) blood vessel to the total augmented area at 2, 4, and 8 weeks in the control and experimental group. (* *p* < 0.05; NS, not significant).

Bone marrow area (measured from 4 weeks onward) in the control group was 0.44 ± 0.17% and 3.82 ± 0.33% at 4 and 8 weeks, respectively. In the experimental group, the values were 2.02 ± 0.26% and 7.12 ± 0.95%. Bone marrow area at 8 weeks was significantly greater than at 4 weeks in both groups (*p* < 0.05). The bone marrow area was significantly larger in the experimental group at 8 weeks compared with the control group (Figure 8a).

Blood vessel area in the control group was 2.68 ± 0.10%, 7.20 ± 0.43%, and 4.59 ± 0.38% at 2, 4, and 8 weeks, respectively. In the experimental group, the values were 5.35 ± 0.12%, 9.14 ± 0.34%, and 6.65 ± 0.47%. Blood vessel area peaked at 4 weeks and was significantly higher than at 2 and 8 weeks in both groups (*p* < 0.05). The blood vessel area was significantly greater in the experimental group at all time points compared with the control group (Figure 8b).

### 3.3. Tartrate-Resistant Acid Phosphatase (TRAP) Staining

**Figure 9:**
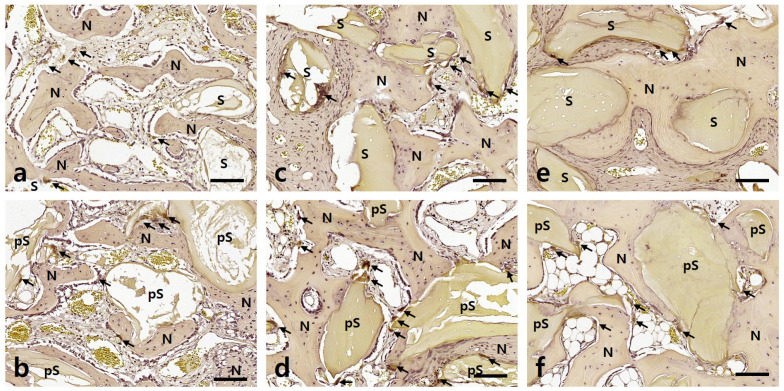
High-magnification images (×200) showing TRAP-stained osteoclasts (arrows) in the control group at 2 weeks (**a**), 4 weeks (**c**), and 8 weeks (**e**), and in the experimental group at 2 weeks (**b**), 4 weeks (**d**), and 8 weeks (**f**). N, newly formed bone; S, SANTA-OSS particles; pS, plasma-treated SANTA-OSS particles (Scale bar: 100 μm).

In the control group, TRAP-positive osteoclasts were observed on the surfaces of newly formed bone and SANTA-OSS particles at 2 weeks (Figure 9a). Their number increased at 4 weeks (Figure 9c) and decreased again at 8 weeks (Figure 9e). In the experimental group, numerous TRAP-positive osteoclasts were already present on newly formed bone and plasma-treated SANTA-OSS surfaces at 2 weeks (Figure 9b). The number was highest at 4 weeks (Figure 9d) and decreased at 8 weeks (Figure 9f).

**Figure 10:**
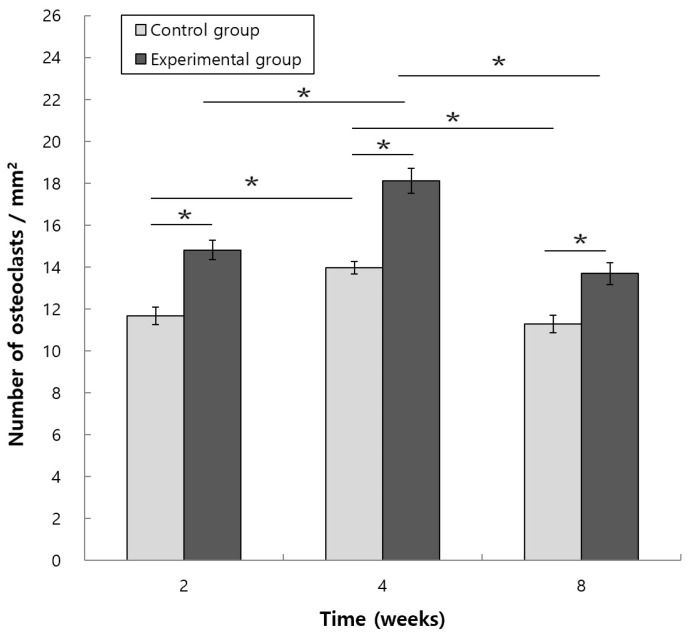
Histomorphometric measurement of the number of TRAP-stained osteoclasts by 1 mm^2^ of the augmented area at 2, 4, and 8 weeks in the control group and experimental group. (* *p* < 0.05).

Quantitatively, the number of TRAP-positive osteoclasts per 1 mm^2^ in the control group was 11.68 ± 0.41, 13.97 ± 0.30, and 11.29 ± 0.41 at 2, 4, and 8 weeks, respectively. In the experimental group, the values were 14.82 ± 0.47, 18.12 ± 0.60, and 13.69 ± 0.53. The number peaked at 4 weeks and was significantly higher than at 2 and 8 weeks in both groups (*p* < 0.05). The number of TRAP-positive osteoclasts was significantly greater in the experimental group at all time points compared with the control group (Figure 10).

## 4. Discussion

Allografts are generally known to possess superior osteoinductive properties compared to xenografts due to their high content of intrinsic growth factors and bone morphogenetic proteins (BMPs). However, there is a potential risk of greater vulnerability to immune responses and bacterial contamination in the oral environment due to saliva and the oral microbiome [9,18]. In contrast, while xenografts exhibit relatively lower osteoinductive properties, they are the most widely used bone substitutes in clinical practice due to their excellent structural stability, low immunogenicity, and virtually unlimited supply, making them the most widely used bone substitute in clinical practice. Xenografts, however, still have inherent long-term limitations, such as slow remodeling speed and incomplete replacement by vital bone [10,19].

In this study, bovine xenografts (SANTA-OSS) were selected, and non-thermal plasma treatment was applied to overcome the limitations in osteoinductivity and bone remodeling speed. In the clinical oral environment, saliva and oral microflora can directly impair bone healing and defect stability by promoting bacterial adhesion and inflammatory responses, which may compromise graft integration [9]. The non-thermal plasma treatment applied in this study, by restoring superhydrophilicity and removing surface contaminants, is expected to mitigate these adverse effects and improve graft performance in the oral cavity [12].

In critical-size defects, spontaneous bone healing is limited and often results in fibrous tissue formation [10]. In the present study, no fibrous or scar tissue formation was observed around the graft particles in either the control or experimental group, indicating favorable graft integration and space-maintaining properties of both untreated and plasma-treated SANTA-OSS. When graft material is combined with blood clot, PRF, or PRP, healing enhances angiogenesis and growth factor delivery, thereby further promoting healing [20]. In the present study, defects were filled with graft material only, without any additional blood products or biological agents, to evaluate the isolated effect of non-thermal plasma treatment.

The experimental results of this study showed that non-thermal atmospheric pressure plasma treatment significantly promotes and enhances new bone formation in a rabbit calvarial defect model. At all observation periods (2, 4, and 8 weeks), the experimental group treated with plasma-treated SANTA-OSS exhibited a larger volume of new bone, faster absorption of the graft material, a wider bone marrow space, and increased angiogenesis compared to the untreated control group. These results suggest that simple plasma treatment using the ACTILINK™ Reborn system in the dental clinic has the potential to significantly improve the osteogenic capacity of bone graft materials [21,22].

In this study, the experimental group demonstrated superior results compared to the control group across all observed indicators; however, the most notable difference was the amount of new bone formation. The plasma-treated group exhibited a significantly higher proportion of new bone compared to the control group throughout all observation periods, and notably, at week 8, the new bone extended to the center of the defect site. This rapid centripetal bone growth is a phenomenon rarely observed within a short period with conventional xenograft materials. Furthermore, the absorption rate of the graft material in the experimental group was faster than that of the control group, and distinct bone marrow spaces containing adipose tissue were observed at week 8. These results suggest that plasma treatment not only promotes bone formation but also induces the balanced remodeling of the graft scaffold, enabling physiological bone marrow formation more rapidly than in the control group [23,24]. The configuration of the bone defect significantly influences the stability of the graft and bone regeneration. While four-wall defects provide the most favorable conditions for predictable healing, the non-contained critical-size calvarial defect used in this study represents a more challenging environment. Despite these demanding conditions, the plasma-treated xenograft demonstrated remarkably enhanced new bone formation and favorable bone remodeling.

In addition, another important finding is that the vascular area in the plasma-treated group significantly increased starting from week 2. Promoting angiogenesis is considered an essential prerequisite for successful bone regeneration because it supplies oxygen, nutrients, and progenitor cells to the defect site [25]. The number of TRAP-positive osteoclasts increased at week 4, supporting the concept that the graft is reformed more actively by plasma treatment. The pattern of osteoclast activity peaking at week 4 and decreasing at week 8 suggests that the stages of bone resorption and bone formation are efficiently linked, which is essential for mature bone development [26].

The biological mechanisms underlying these improvements are closely related to the well-documented effects of non-thermal plasma on biomaterial surfaces [27,28]. Plasma effectively removes hydrocarbon contaminants that accumulate during storage (biological aging), restores superhydrophilicity, and markedly enhances protein adsorption (fibronectin, albumin, etc.) [27]. These surface changes promote early cell attachment, proliferation, and differentiation of osteogenic cells while reducing oxidative stress and improving hemocompatibility [28]. Although the present study focused on histomorphometry (Part I), the accelerated vascularization and osteoclast activity observed here strongly suggest that plasma treatment creates a more favorable microenvironment for both osteogenesis and angiogenesis—findings that will be further elucidated by immunohistochemical analysis of osteogenic and angiogenic markers in the companion Part II study.

When compared with previous animal studies using biological enhancers, the magnitude of improvement observed in the present study is comparable to that reported by Kim et al. (2025), who demonstrated significantly enhanced new bone formation and graft resorption using mesenchymal stem cell-conditioned media combined with human allogeneic bone in the same rabbit calvarial defect model [15]. In addition, these results are consistent with Tallarico et al. (2025), who reported that vacuum plasma surface treatment significantly improves the hydrophilicity and wettability of bone graft substitutes and resorbable membranes [17]. However, unlike conditioned media which requires complex preparation and raises concerns regarding cost and regulatory issues, the plasma activation used in this study is a simple, immediate chairside physical method that requires no additional biological agents [29]. This suggests that non-thermal plasma treatment may offer a more practical and reproducible alternative for enhancing xenograft performance.

Nevertheless, several limitations should be acknowledged. First, this study evaluated healing only up to 8 weeks. Although significant differences between groups were already observed at this point, complete replacement of the graft, the long-term stability of regenerated bone volume, and the potential for bone resorption in later stages could not be evaluated. Future studies with extended observation periods (12–24 weeks) are warranted to confirm the long-term behavior and bone volume stability of plasma-treated xenografts. Second, since this study was conducted in a non-loaded calvarial defect model, the results cannot be directly applied to load-bearing alveolar ridge or sinus augmentation sites in humans. Finally, while histomorphometric analysis provides reliable quantitative data on bone volume and remodeling, it does not fully elucidate the underlying cellular and molecular mechanisms. This will be addressed in the forthcoming immunohistochemical study. Taken together, the present findings indicate that plasma treatment significantly enhances the bone regenerative capacity of SANTA-OSS.

## 5. Conclusions

Within the limitations of this study, non-thermal atmospheric pressure plasma treatment of SANTA-OSS significantly enhanced new bone formation, accelerated graft resorption, promoted bone marrow development, and increased vascularization in the rabbit calvarial defect model. Plasma activation using the ACTILINK™ Reborn system, performed simply chairside, can significantly enhance the osteogenic capacity of bovine cancellous bone grafts without the need for additional biological agents. Plasma-treated xenografts offer more predictable and rapid bone regeneration, possessing the potential to accelerate implant placement and improve clinical outcomes in alveolar bone augmentation. Further mechanistic insights will be presented in the upcoming immunohistochemistry study.

## Data Availability

The original contributions presented in this study are included in the article. Further inquiries can be directed to the corresponding author.

## References

[1] Findler M., Chackartchi T., Rimbach S., Mann J., Tobias G. (2026). Clinical Success Rates of Dental Implants with Bone Grafting in a Large-Scale National Dataset. J. Funct. Biomater..

[2] Elboraey M.O., Alqutaibi A.Y., Aboalrejal A.N., Borzangy S., Zafar M.S., Al-Gabri R., Alghauli M.A., Ramalingam S. (2025). Regenerative approaches in alveolar bone augmentation for dental implant placement: Techniques, biomaterials, and clinical decision-making: A comprehensive review. J. Dent..

[3] Yu S.H., Saleh M.H.A., Wang H.L. (2023). Simultaneous or staged lateral ridge augmentation: A clinical guideline on the decision-making process. Periodontology 2000.

[4] Zhao R., Yang R., Cooper P.R., Khurshid Z., Shavandi A., Ratnayake J. (2021). Bone Grafts and Substitutes in Dentistry: A Review of Current Trends and Developments. Molecules.

[5] Wang H., Kang J. (2026). Bone grafts and synthetic substitutes in dental applications. Front. Bioeng. Biotechnol..

[6] Ferraz M.P. (2023). Bone Grafts in Dental Medicine: An Overview of Autografts, Allografts and Synthetic Materials. Materials.

[7] Nicolae C.L., Pîrvulescu D.C., Niculescu A.G., Epistatu D., Mihaiescu D.E., Antohi A.M., Grumezescu A.M., Croitoru G.A. (2024). An Up-to-Date Review of Materials Science Advances in Bone Grafting for Oral and Maxillofacial Pathology. Materials.

[8] Heimes D., Pabst A., Becker P., Hartmann A., Kloss F., Tunkel J., Smeets R., Kämmerer P.W. (2024). Comparison of morbidity-related parameters between autologous and allogeneic bone grafts for alveolar ridge augmentation from patients’ perspective-A questionnaire-based cohort study. Clin. Implant. Dent. Relat. Res..

[9] Ciszyński M., Dominiak S., Dominiak M., Gedrange T., Hadzik J. (2023). Allogenic Bone Graft in Dentistry: A Review of Current Trends and Developments. Int. J. Mol. Sci..

[10] Zhang J., Zhang W., Yue W., Qin W., Zhao Y., Xu G. (2025). Research Progress of Bone Grafting: A Comprehensive Review. Int. J. Nanomed..

[11] Makary C., Menhall A., Lahoud P., Yang K.R., Park K.B., Razukevicius D., Traini T. (2024). Bone-to-Implant Contact in Implants with Plasma-Treated Nanostructured Calcium-Incorporated Surface (XPEEDActive) Compared to Non-Plasma-Treated Implants (XPEED): A Human Histologic Study at 4 Weeks. Materials.

[12] Schafer S., Swain T., Parra M., Slavin B.V., Mirsky N.A., Nayak V.V., Witek L., Coelho P.G. (2024). Nonthermal Atmospheric Pressure Plasma Treatment of Endosteal Implants for Osseointegration and Antimicrobial Efficacy: A Comprehensive Review. Bioengineering.

[13] Fischer M., Bortel E., Schoon J., Behnke E., Hesse B., Weitkamp T., Bekeschus S., Pichler M., Wassilew G.I., Schulze F. (2023). Cold physical plasma treatment optimization for improved bone allograft processing. Front. Bioeng. Biotechnol..

[14] Barausse C., Tayeb S., Pellegrino G., Sansavini M., Mancuso E., Mazzitelli C., Felice P. (2025). Cold Plasma Treatment on Titanium Implants and Osseointegration: A Systematic Review. Appl. Sci..

[15] Kim Y.-K., Choi H., Kim H.-G., Sohn D.-S. (2025). Impact of Plasma Surface Treatment on Implant Stability and Early Osseointegration: A Retrospective Cohort Study. Materials.

[16] Stacchi C., Rapani A., Montanari M., Martini R., Lombardi T. (2025). Effect of Vacuum Plasma Activation on Early Implant Stability: A Single-Blind Split-Mouth Randomized Clinical Trial. J. Oral. Maxillofac. Res..

[17] Tallarico M., Meloni S.M., Troia M., Cacciò C., Lumbau A.I., Gendviliene I., Ceruso F.M., Pisano M. (2025). The Use of Vacuum Plasma Surface Treatment to Improve the Hydrophilicity and Wettability of Bone Graft Substitutes and Resorbable Membranes: An In Vitro Study. Dent. J..

[18] Moraschini V., de Almeida D.C.F., Calasans-Maia M.D., Kischinhevsky I.C.C., Louro R.S., Granjeiro J.M. (2020). Immunological response of allogeneic bone grafting: A systematic review of prospective studies. J. Oral. Pathol. Med..

[19] Rodriguez A.E., Nowzari H. (2019). The long-term risks and complications of bovine-derived xenografts: A case series. J. Indian Soc. Periodontol..

[20] Miron R.J., Fujioka-Kobayashi M., Bishara M., Zhang Y., Hernandez M., Choukroun J. (2017). Platelet-Rich Fibrin and Soft Tissue Wound Healing: A Systematic Review. Tissue Eng. Part B Rev..

[21] Wu C., Ma K., Zhao H., Zhang Q., Liu Y., Bai N. (2020). Bioactive effects of nonthermal argon-oxygen plasma on inorganic bovine bone surface. Sci. Rep..

[22] Shimatani A., Toyoda H., Orita K., Hirakawa Y., Aoki K., Oh J.S., Shirafuji T., Nakamura H. (2021). In vivo study on the healing of bone defect treated with non-thermal atmospheric pressure gas discharge plasma. PLoS ONE.

[23] Ahn J.-J., Yoo J.-H., Bae E.-B., Kim G.-C., Hwang J.J., Lee W.-S., Kim H.-J., Huh J.-B. (2019). The Effects of Atmospheric Pressure Argon Plasma Treated Bovine Bone Substitute on Bone Regeneration. Coatings.

[24] Akçay H., Ercan U.K., Bahçeci S., Ulu M., Ibiş F., Enhoş Ş. (2020). The Effect of Atmospheric Pressure Cold Plasma Application on Titanium Barriers: A Vertical Bone Augmentation. J. Craniofac. Surg..

[25] Arndt S., Unger P., Berneburg M., Bosserhoff A.-K., Karrer S. (2018). Cold atmospheric plasma (CAP) activates angiogenesis-related molecules in skin keratinocytes, fibroblasts and endothelial cells and improves wound angiogenesis in an autocrine and paracrine mode. J. Dermatol. Sci..

[26] Xiang Q., Li L., Ji W., Gawlitta D., Walboomers X.F., van den Beucken J.J.J.P. (2024). Beyond resorption: Osteoclasts as drivers of bone formation. Cell Regen..

[27] Zheng Z., Ao X., Xie P., Wu J., Dong Y., Yu D., Wang J., Zhu Z., Xu H.H.K., Chen W. (2020). Effects of novel non-thermal atmospheric plasma treatment of titanium on physical and biological improvements and in vivo osseointegration in rats. Sci. Rep..

[28] Kahm S.H., Lee S.H., Lim Y., Jeon H.J., Yun K.-I. (2024). Osseointegration of Dental Implants after Vacuum Plasma Surface Treatment In Vivo. J. Funct. Biomater..

[29] Nonnenmacher L., Fischer M., Haralambiev L., Bekeschus S., Schulze F., Wassilew G.I., Schoon J., Reichert J.C. (2023). Orthopaedic applications of cold physical plasma. EFORT Open Rev..

